# NGS-basierte („next generation sequencing“) molekulare Panelanalyse des metastasierten Prostatakarzinoms: Wie häufig finden wir therapierbare Mutationen? NGS beim metastasierten Prostatakarzinom

**DOI:** 10.1007/s00120-024-02493-2

**Published:** 2025-01-21

**Authors:** Olivia Steenbock, Pia Paffenholz, Constantin Rieger, Julian Heidenreich, David Pfister, Melanie von Brandenstein, Axel Heidenreich

**Affiliations:** 1 Klinik für Urologie, Uro-Onkologie, roboter-assistierte und spezielle urologische Chirurgie, Uniklinik Köln, Kerpener Str. 62, 50927 Köln, Deutschland; 2 Institut für Pathologie, Uniklinik Köln, Köln, Deutschland; 3 Klink für Urologie, Medizinische Universität Wien, Wien, Österreich

**Keywords:** Androgendeprivation, PARP-Inhibitoren, Kastrationsresistentes Prostatakarzinom, Personalisierte Medizin, Zielgerichtete Therapie, Molekulare Diagnostik, Androgen deprivation, PARP inhibitors, Castration resistant prostate neoplasms, Personalized medicine, Targeted therapy, Molecular diagnostics

## Abstract

**Einleitung:**

Die Leitlinien fordern nach Versagen der systemischen Ersttherapie des metastasierten hormonsensitiven Prostatakarzinoms (mHSPC) eine molekulare Analyse zur Identifikation therapierbarer Mutationen. Wir berichten über unsere Ergebnisse der molekularen Diagnostik bei Patienten mit metastasiertem kastrationsresistenten PCA (mCRPC).

**Patienten und Methodik:**

311 Patienten mit mCRPC erhielten eine molekulare Panelanalyse von archivierten Prostatektomiepräparaten oder Computertomografie(CT)-gestützten Biopsien progredienter Metastasen mittels standardisiertem NGS-Verfahren eines Panels von 18 spezifischen Mutationen bzw. dem TSO500-Panel.

**Ergebnisse:**

Unabhängig vom Entnahmeort hatten 299/311 (96 %) der Biopsien einen ausreichenden DNA-Gehalt für das NGS. NGS erfolgte aus Prostata (31 %), Lymphknoten (26 %), viszeralen (17 %) und ossären (18 %) Metastasen. Bei 223 (75 %) bzw. 76 (25 %) Patienten wurden aktivierende/inhibierende bzw. keine Mutationen identifiziert. Am häufigsten fanden sich Mutationen der HRD-Gene (BRCA 1/2, ATM, CDK12, CHEK2, FANCA, Rad51C) sowie des p53 mit jeweils 22 %. Die Mehrzahl der p53Mutationen waren inaktivierend, in 3 Fällen wurde eine Gain-of-function-Mutation identifiziert. Mutationen der HRD-Gene inklusive eines positiven HRD-Scores waren in > 50 % pathogen, so dass PARP-Inhibitoren eingesetzt werden konnten. Aktivierende Androgenrezeptor – sowie inaktivierende PTEN/aktivierende PIC3Ca-Mutationen fanden sich bei 42 (14 %) bzw. 24 (8 %) Patienten. Aufgrund der spezifischen AR-Mutationen wurde eine Therapieumstellung bei 14 Patienten vorgenommen. Mutationen der Mismatch-repair-deficiency-Gene/MSI-high lagen in 3 Fällen vor, so dass Pembrolizumab appliziert werden konnte. Die Addition des TSO500-Panels identifizierte nur bei 4,5 % der Patienten zusätzliche Mutationen, bei nur 2 % der Patienten hätte diese eine therapeutische Implikation gehabt.

**Schlussfolgerungen:**

Eine NGS-Analyse beim mCRPC zeigt bei einem Drittel der Patienten Mutationen auf, die bereits jetzt zielgerichtet therapierbar sind. Eine fundierte Analyse der HRD-Gene sowie von AR-Mutationen sollte nach Versagen der Erstlinientherapie erfolgen. Eine ausgedehnte molekulare Analyse empfiehlt sich nach Versagen der sequentiellen Standardtherapie. Die molekulare Analyse mittels des TSO500-Panels ist nur in wenigen Fällen zielführend.

**Graphic abstract:**

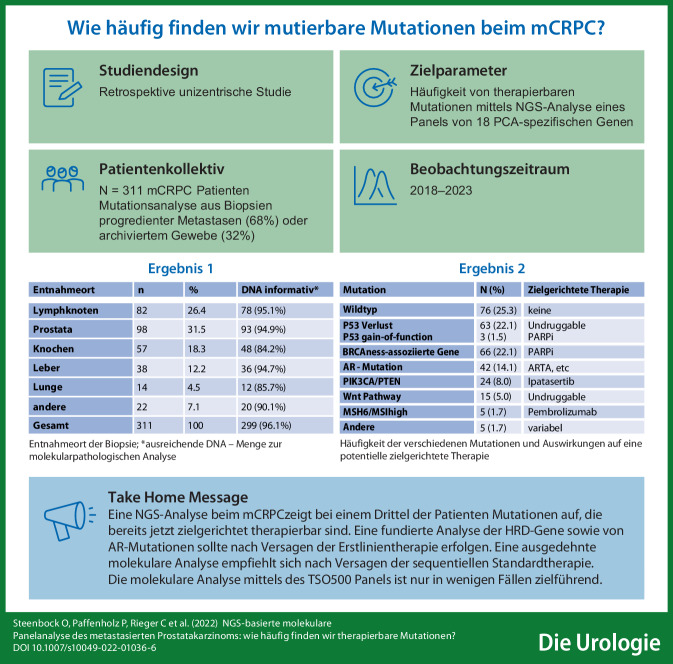

## Abstract (en)

**Introduction:**

Prostate cancer guidelines recommend molecular analysis of biomaterial following resistance to first-line systemic therapy in order to identify druggable mutations. We report on our results of molecular analysis of tissue specimens via next generation sequencing (NGS) in men with metastatic castration resistant prostate cancer (mCRPC).

**Patients and methods:**

In all, 311 mCRPC patients underwent NGS analysis from biopsy samples of progressive metastatic lesions or archival radical prostatectomy specimens. NGS analysis was either performed using a panel of 18 prostate cancer-specific amplicons or via the TS0500 panel.

**Results:**

Of the 311 biopsies, 299 (96%) revealed sufficient DNA content for NGS analysis independent on the specimen origin. Biopsies were taken from prostate (31%), lymph nodes (26%), visceral (17%) or osseous (18%) metastases. In 223 (75%) and 76 (25%) patients activating/inhibiting and no mutations were identified, respectively. Most frequently, mutations of HRD genes including a positive HRD score and p53 were identified in 22% of patients each. About 50% of HRD gene mutations were pathogenic and treatment with PARP inhibitors was initiated. Although the majority of p53 alterations were inactivating mutations, 3 patients demonstrated gain-of-function mutations resulting in an inactivation of ATM. Activating androgen receptor mutations and inactivating PTEN mutations were identified in 42 (14%) and 24 (8%) patients, respectively. Specific AR mutations resulted in a switch of hormonal therapy. Mutations of mismatch repair deficiency genes/MSI high were identified in 5 patients resulting in the administration of pembrolizumab. Addition of the TSO 500 panel identified additional mutations in 4.5% of patients and only 2% of the total cohort would have benefitted from this large panel with the identification of druggable mutations.

**Conclusion:**

Druggable mutations were identified in one third of mCRPC patients using an 18 amplicon-panel analyzed via NGS. Based on our data, molecular analysis of AR mutations or mutations of the HRD genes should be performed following progression after first-line hormonal therapy. A more extensive molecular analysis seems to be useful following progression to the standard sequential hormonal, cytotoxicand radioligand therapies. Use of the expensive TSO 500 panel seems to be of additional therapeutic value in only a minority of patients.

## Einleitung

Das Prostatakarzinom (PCA) ist in Deutschland mit jährlich ca. 78.000 Neudiagnosen der häufigste solide Tumor des Mannes [1]. Auch wenn sich initial < 10 % der Patienten mit einem primär metastasierten PCA präsentieren, entwickeln bis 40 % aller Patienten nach lokaler Therapie Metastasen [2]. In der Situation des systemisch metastasierten hormonsensitiven PCA (mHSPC) stellt die Androgendeprivation (ADT) in Kombination mit einem modernen Androgenrezeptorbiosyntheseinhibitor (ARI) bei geringer Metastasenlast oder in Kombination mit einem ARI und Docetaxel bei hoher Metastasenlast und Eignung für Docetaxel die leitlinienbasierte Therapie der Wahl dar [3, 4]. Lediglich bei Patienten mit geringer Metastasenlast sollte zusätzlich zur systemischen Therapie eine lokale Therapie des Primarius addiert werden [5–7]. Trotz einer hohen Ansprechrate werden alle Patienten mit einem mHSPC ein kastrationsresistentes PCA (CRPC) entwickeln, das trotz der modernen Behandlungsmöglichkeiten durch molekulare Ansätze oder die Radioligandentherapie durch einen aggressiven Verlauf und eine limitierte Überlebenszeit charakterisiert ist.

Somatische Mutationen der Karzinomzellen führen zu Funktionsveränderungen spezifischer Gene oder Signaltransduktionskaskaden, die in die molekulare Pathogenese, Metastasierung und Resistenzentwicklung involviert sind [8]. Die Identifikation solcher genomischer Alterationen kann dazu beitragen, die Mechanismen der Tumorprogression zu verstehen und innovative Therapieoptionen zu entwickeln.

Verschiedene Wege der molekularen Diagnostik beim mHPSC und noch mehr beim mCRPC haben zu der Identifikation von Treibermutationen geführt, die in den Prozess der Progression involviert sind und zum Teil bereits therapeutisch gezielt angegangen werden können [9, 10]. Jedoch ist die molekulare Diagnostik des mHSPC bzw. des mCRPC in der klinischen Routine herausfordernd, da das zur Verfügung stehende Material meist limitiert und archiviert ist und die wenigsten Patienten in der klinischen Routine eine Biopsie progedienter Metastasen erhalten [9, 10].

Es ist die Zielsetzung unserer retrospektiven Analyse, die Möglichkeiten der molekularen Diagnostik einer Panelanalyse von verschiedenen Mutationen in Metastasengewebe auf der Basis des modernen „next generation sequencing“ (NGS) und der sich daraus potenziell ergebenden therapeutischen Konsequenzen im klinischen Alltag aufzuzeigen.

## Patienten und Methodik

Es wurden 311 Patienten mit einem mCRPC aus unserer Datenbank identifiziert, bei denen zwischen 2018 und 2023 eine molekularpathologische Untersuchung zur Identifikation therapierbarer Mutationen aus frisch gewonnenen Biopsien progredienter Metastasen bzw. archivierter, paraffin-eingebetteter radikaler Prostatektomie- oder Biopsiepräparate durchgeführt wurde.

Alle Patienten erfüllten die Kriterien der EAU-Leitlinien zur Definition eines mCRPC [4]. Bei allen Patienten erfolgte zur Identifikation progredienter und möglicher biopsierbarer Metastasen eine konventionelle Bildgebung mittels eines Computertomografie(CT)-Thorax, Abdomen und Becken sowie eine molekulare Bildgebung mit einem ^68^Ga-PSMA oder einem ^18^F‑1007-PSMA-PET/CT (Positronenemmissionstomografie). Die Frage nach einer möglichen CT-gestützten Biopsie progredienter Metastasen wurde interdisziplinär mit den interventionellen Radiologen der Klinik diskutiert. Waren die Metastasen unabhängig von ihrer Lokalisation biopsierbar, wurden 1–3 Stanzzylinder in Lokalanästhesie unter stationären bzw. ambulanten Bedingungen entnommen. Im Falle eines negativen bildgebenden Befunds oder nicht biopsierbarer Metastasen wurde archiviertes Gewebe, meist in Form von radikalen Prostatektomiepräparaten, analysiert.

Das Material wurde vor weiterer Aufarbeitung durch einen erfahrenen Uropathologen befundet, um die tumortragenden Areale für die nachfolgende Mikrodissektion zu markieren.

## DNA-Extraktion aus archiviertem Gewebe

Von dem archivierten Material wurden 10-mm-Schnitte angefertigt und der tumortragende Anteil wurde von einem Uropathologen markiert. Der markierte Bereich wurde mit einem Skalpell vom Glasträger abgekratzt und in ein 1,5-ml-Röhrchen überführt. Für die Extraktion wurde das QiAamp DNA FFPE Advanced Kit (Qiagen, Hilden, Deutschland) nach Herstellerangaben verwendet. Nach Lösung der DNA von der Qiagen-Säule wurde die Quantifizierung sowie die Kontrolle der Reinheit mittels NanoDrop (Thermo Fisher Scientific, Langerwehe, Deutschland) durchgeführt. Um eine mögliche Degradierung der DNA auszuschließen, wurden die DNA-Proben stichpunktartig in einem 1 %-Agarose-Gel geladen.

### Molekulare Analyse aus Biopsien

Im Falle von CT-gestützten Biopsien wurden 1–3 Gewebeproben aus der Zielläsion entnommen, in Kochsalzlösung asserviert und unmittelbar in das Institut für Pathologie verbracht, nach einem standardisierten Protokoll aufgearbeitet und analysiert (s. unten).

### „Gene panel analysis by parallel sequencing“

Mit dem Qubit dsDNA HS Assay (Thermo Fisher Scientific) auf dem Qubit 2.0 Fluorometer (Thermo Fisher Scientific) wurden 40 ng DNA quantifiziert und nachfolgend auf dem Covaris E220 Focused-ultrasonicator (Woburn, MA, USA) mit der 8 microTUBE-50 Strip AFA Fiber V2 entsprechend des Herstellermanuals „sheared“. Die Behandlungszeit der Proben ist optimiert für FFPE-Material und das Setting ist wie folgt: Peak Incident Power (W): 75; Duty Factor: 15 %; Cycles per Burst: 500; Treatment Time (s): 360; Temperature (°C): 7; Water Level: 6. Für die DNA library Präparation und die Anreicherung wurde der TruSight Oncology 500 Kit (Illumina) entsprechend des Herstellermanuals verwendet. Die Libraries wurden nach Anreicherung quantifiziert, gepoolt und auf einem NextSeq 500 (Illumina, San Diego, CA, USA) sequenziert.

Die Qualität der NextSeq 500 (Illumina)-Sequenzen wurde auf dem Illumina Sequencing Analysis Viewer (Illumina) beurteilt. Die Sequenzierungsdaten wurden mit der TruSight Oncology 500 Local App Version 1.3.0.39 (Illumina) analysiert. Neben den reinen Mutationsdaten konnten der Mikrosatellitenstatus sowie die „tumor mutational burden“ analysiert werden.

### Expressionsanalyse

Die Differentialexpression der selektierten Gene auf RNA-Ebene wurde mittels der Anwendung eines kommerziellen NanoString Panel (NanoString Technologies, Inc., Seattle, WA, USA) durchgeführt. Die isolierte mRNA wurde mit einem Set von genspezifischen und fluoreszenzmarkierten Gensonden für 18 h bei 65 °C hybridisiert. Die Hybridisationsprodukte wurden weiter auf einem nCounter PrepStation (NanoString Technologies, Inc.) präpariert, bevor diese in die entsprechenden Cartridges geladen wurden. Das digitale Zählen der Fluoreszenzsignale erfolgte auf dem nCounter Digital Analyzer. Nachfolgend wurde die Datenanalyse inklusive der erforderlichen Statistik mit der nsolver 3.0 Software und dem zugehörigen Advanced Analysis 2.0 Package durchgeführt. Die Normalisierung der Proben erfolgt mittels Analyse der in dem Panel inkludierten Housekeeping-Gene.

Die Mutationsanalyse erfolgte initial mittels NGS [22] in einem von uns zusammengestellten „Prostatakarzinompanel“ von 18 Multiplex PCR Amplikons (AR, ATM, AURKA/MYC, BRCA 1/2, CDK12, CTNNB1, DLL3, ETS family, FOXA1, FOX01, MED12, PIK3CA, PTEN, RAD51C, TP53, Wnt-Pathway), deren Generierung mit einem GeneRead DNAseq Custom Panel V2 (Qiagen) durchgeführt wurde. Das Panel wurde generiert, um die häufigsten, potenziell therapierbaren Mutationen in einer Analyse nachweisen zu können. Zudem haben wir den MSI-Status und den HRD-Score berechnet (s. unten). Ab 2023 führen wir in ausgewählten Fällen die NGS-Analyse mittels des TSO500-Panels durch. Für die Library-Erstellung wurden GeneRead DNA Library I Core, GeneRead DNA I Amp Kits sowie NEXTflex-96 DNA Barcode Adapter verwendet.

Die Analyse der Defizienz der homologen Rekombinationsreparatur (HRD) erfolgt über die Kombination von BRCA 1- und BRCA 2*-*Mutationen sowie dem HRD-Score (Genomic ScarScore GSS) mittels des HANDLE HRD Focus Panels (Halo-shape Annealing und Defer-Ligation Enrichment, AmoyDx). Zur gezielten Erfassung von Varianten und HRD-Score wurde mittels Molecular Inversion Probe (MIP)-Technologie eine Parallelsequenzierungsbibliothek hergestellt. Die Sequenzierung wurde auf dem NextSeq (Illuma) durchgeführt. Die anschließende Datenauswertung erfolgte mit dem ANDAS Server (AmoyDx). Die Datenauswertung erfolgte mit den folgenden Schwellenwerten: Tumorzellgehalt ≥ 30 %, GScore positiv bei ≥ 50, HRD positiv: GScore bei ≥ 50 oder BRCA 1/2-Kategorie-4/5 Mutation.

Zudem führten wir neben der NGS-basierten Analyse des Mikrosatellitenstatus (MSIhigh) die immunhistochemische Expressionsanalyse der Mismatch-repair-Gene MSH2, MSH6, PMS2 und MLH1 durch.

Basierend auf den molekularen Alterationen der NGS-Analyse wurden die folgenden Datenbanken gesichtet, um bereits beschriebene mutationsbasierte Therapieoptionen anderer Tumoren zu identifizieren: OncoKB (http://oncokb.org), Clin Var (http://www.ncbi.nlm.nih.gov/clinvar/), JAX-CKB (https://ckb.jax.org), COSMIC (https://cancer.sanger.ac.uk/cosmic) sowie My Cancer Genome (https://www.mycancergenome.org).

## Ergebnisse

Es wurden Gewebeproben unterschiedlicher Lokalisationen von 311 Patienten mit einem mCRPC zur weitergehenden molekularpathologischen Analyse gewonnen (Tab. 1). Bei 82 Proben handelte es sich um archiviertes Material, 229 Proben wurden durch Biopsie progredienter Metastasen gewonnen. Alle Biopsien wurden CT-gestützt unter Lokalanästhesie durchgeführt und bei keinem der 229 Patienten kam es zu signifikanten interventionsbedingten Komplikationen. 299/311 (96 %) der Proben wiesen unabhängig vom Entnahmeort eine ausreichende DNA-Menge zur weitergehenden Analyse auf.

**Tab. 1:** Entnahmeort der Biopsie

Entnahmeort	*n*	%	DNA informativ^a^ (%)
Lymphknoten	82	26,4	78 (95,1)
Prostata	98	31,5	93 (94,9)
Knochen	57	18,3	48 (84,2)
Leber	38	12,2	36 (94,7)
Lunge	14	4,5	12 (85,7)
Andere	22	7,1	20 (90,1)
*Gesamt*	*311*	*100*	*299 (96,1)*

^a^Ausreichende DNA-Menge zur molekularpathologischen Analyse

Das mediane Alter der Patienten betrug 70,3 (45–89) Jahre.

Die mediane Serumkonzentration für PSA und Testosteron betrug 98,7 (14,3–1897) ng/ml bzw. 26,5 (12–36,5) ng/dl.

Die Patientencharakteristika inklusive der Vortherapien und des Zeitpunktes der molekularen Testung sind in Tab. 2 dargestellt (Tab. 3).

**Tab. 2:** Patientencharakteristika

Parameter	*n* (%)
Medianes Alter (Jahre)	70,3 (45–89)
Medianes PSA (ng/ml)	98,7 (14,3–1897)
Medianes Testosteron (ng/dl)	26,5 (12,0–36,5)
*ECOG Performance Status*	
ECOG 1	140 (45,0)
ECOG 2	155 (49,8)
ECOG 3	016 (5,1)
Lokaltherapie (RT/RPE)	224 (72,0)
De-novo-Metastasierung	087 (18,0)
*Initiales Stadium bei Metastasierung*	
M1a	031 (10)
M1b	140 (45)
M1c	047 (15)
Kombination	093 (30)
*Vortherapie*	
Gruppe 1	26 (8,4)
Gruppe 2	21 (6,8)
Gruppe 3	15 (4,8)
Gruppe 4^*^	208 (66,9)
Gruppe 5^a^	–

*Gruppe 1* ADT ≥ Docetaxel ≥ ARTA ≥ Cabazitaxel, *Gruppe 2* ADT ≥ ARTA ≥ Docetaxel ≥ Cabazitaxel, *Gruppe 3* wie 1 + 2 + Radium 223, *Gruppe 4* ADT + ARTA ≥ Docetaxel + ^177^Lu-PSMA, *Gruppe 5* ADT + ARTA ≥ Docetaxel

^a^Testung bereits nach Versagen ADT + ARTA

**Tab. 3:** Funktion, Mutationen und Therapiemöglichkeiten der am häufigsten diagnostizierten Mutationen in der retrospektiven Analyse

Gen	Funktion	Mutation	Therapie
P53	Tumorsuppressorgen; Regulation des Zellzyklus; Induktion der Apoptose; DNA-Reparatur	–	Aktuell keine spezifische Medikation verfügbar
*BRCAness-Gene*			
BRCA 1/2	Tumorsuppressorgen; Reparatur von DNA-Doppelbrüchen	–	PARP-Inhibitor
ATM	Kodiert die Serin-Proteinkinase ATM; Induktion von Signalkaskaden zur Synthese von DNA-Reparaturproteinen	–	PARP-Inhibitor
RAD51C (BRCA 3)	Tumorsuppressorgen; Reparatur von DNA-Doppelbrüchen	–	PARP-Inhibitor; Cisplatin-basierte Chemotherapie
Androgenrezeptor	Transkriptionsfaktor für die Ausprägung des männlichen Phänotyps Überexpression kann zu Androgenresistenz und Proliferation von Tumorzellen führen		
L702H	Resistenz gegen Bicalutamid, Abirateron	PCA-Proliferation trotz AR-Blockade	Apalutamid, Darolutamid, Enzalutamid
T878A	Resistenz gegen Apa, Bic, Enza, Flu; Prednison als partieller Agonist	PCA-Proliferation trotz AR-Blockade	Abi + Dexamethason; Darolutamid
*p*.875Y	APA und Enza als Agonist; Östradiol als Agonist	PCA-Proliferation trotz AR-Blockade	Darolutamid
*p*.V716M	Darolutamid als Agonist	PCA-Proliferation trotz AR-Blockade	Abi + Pred.; Enzalutamid
W742W/L	Resistenz gegen Bic, Flu	PCA-Proliferation trotz AR-Blockade	Apalutamid, Darolutamid, Enzalutamid
F877L	Apa und Enza als partielle Agonisten	PCA-Proliferation trotz AR-Blockade	Bicalutamid, Darolutamid
T878S	Apa, Bic, Enza, Flu als partielle Antagonisten	PCA-Proliferation trotz AR-Blockade	Abi + Pred; Darolutamid
PIK3Ca	Intrazelluläre Regulation von Wachstum, Proliferation und Metabolismus	Hemmung der Apoptose, Aktivierung der Proteintranslation, Steigerung der Proliferation	Ipatasertib
PTEN	Tumorsuppressorgen Aktivierung der Apoptose	Überschießendes Zellwachstum	Keine zielgerichtete Therapieoption
Wnt-Gene	Essentieller Signaltransduktionsweg für die Embryogenese	Inhibierung des TSG APC ≥ intrazelluläres *β*−Catenin↑ ≥ Zellwachstum	Keine zielgerichtete Therapie verfügbar
MSH6	Bildet mit MSH2 einen Mismatch-Reparaturkomplex, der an fehlerhafte Basenpaare der DNA bindet	Vermehrung von Zellen trotz fehlerhafter DNA ≥ Krebs, Lynch Syndrom	Pembrolizumab
MSI-high	Auftreten neuer Allele innerhalb kurzer, repetitiver DNA-Sequenzen (Mikrosatelliten) und die daraus resultierenden Längenveränderungen als Folge defekter DNA-Reparaturproteine	Bei Defekten der MMR-Gene häufen sich die Mutationen in den DANN-Strängen ≥ Krebs	Pembrolizumab

## Ergebnisse der NGS-Analyse

Insgesamt wiesen 157 (50,5 %) Patienten keine Mutationen bzw. nicht therapierbare Mutationen auf, während sich bei 154 (49,5 %) Patienten Mutationen fanden, die prinzipiell einer zielgerichteten Therapie zugeführt werden könnten (Tab. 4).

Beim Vergleich der durch die NGS-Analyse unseres „hauseigenen“ PCA-Panels ermittelten Mutationen gegenüber der NGS mit dem TSO500-Panel wird deutlich, dass der informative Zugewinn gering ist. Mit dem TSO500-Panel haben wir nur bei 4,5 % der Patienten eine zusätzliche Mutation diagnostiziert, die wiederum bei 2 % der Patienten eine therapeutische Implikation nach sich gezogen hätte.

**Tab. 4:** Häufigkeit der verschiedenen Mutationen und Auswirkungen auf eine potenzielle zielgerichtete Therapie

Mutation	*n* (%)	Zielgerichtete Therapie
Wildtyp	76 (25,4)	Keine
P53-Verlust	66 (22,1)	Undruggable
BRCAness	66 (22,1)	PARPi
AR-Mutation	42 (14,1)	ARTA etc.
PIK3CA/PTEN	24 (8,0)	Ipatasertib
Wnt Pathway	15 (5,0)	Undruggable
MSH6/MSI-high	5 (1,7)	Pembrolizumab
Andere (s. Tab. 9)	5 (1,7)	Tab. 3

### BRCAness-Gene

Eine Mutation der BRCAness-Gene wurde bei 66 (22,1 %) Patienten nachgewiesen, ein positiver HRD-Score wurde bei weiteren 8 (2,7 %) Patienten identifiziert. Am häufigsten fanden sich Mutationen für ATM sowie BRCA 1/2 (Tab. 5), während die Mutationen anderer HRD-Gene bis auf RAD51C eher selten waren. Es wurden 34 unterschiedliche Mutationen identifiziert, von denen 17 (50 %) pathogen waren und damit einer zielgerichteten Therapie zugänglich waren. Unter diesen therapierbaren Mutationen haben wir *n* = 7 identifiziert, die in den von uns genutzten Datenbanken unbekannt waren, jedoch in allen Fällen zu einer inaktivierenden Trunkierung von ATM oder BRCA 1/2 führten. Da alle in dieser Region bisher beschriebenen trunkierenden Mutationen pathogen waren, sind wir davon ausgegangen, dass auch für diese von uns beschriebenen Mutationen eine PARP-Inhibitortherapie Erfolg versprechend sein könnte. 7 (10,6 %) Patienten wiesen eine bisher unbekannte Mutation auf, so dass deren Funktion und somit Zugänglichkeit für eine zielgerichtete Therapie unklar ist. 6 (9,1 %) Patienten zeigten eine Mutation unklarer Signifikanz, die keiner weitergehenden Therapie zugeführt werden kann.

**Tab. 5:** Mutationen und therapeutische Implikationen der BRCAness-Gene

Gen	Exon	Mutation	NG Change-Protein	NG Allelfrequenz	Funktion
ATM	17	*c*.2480A > *G*	*p*.K827R	90,97	Bisher unbekannt
	21	*c*.3153 + 3G > T	*c*.3153 + 3G > T	8,23	Mutation in Splice site, Funktion unbekannt
	24	*c*.3575delA	K1191Rfs3	32,52	*Trunkierend* *=* *pathogen*
	26	*c*.3925G > A	*p*.A1309T	49,98	Mutation uS
	29	*c*.4424A > G	*p*.Y1475C	51,03	Mutation uS
	39	*c*.5890A > G	*p*.K1964E	64,73	Mutation uS
	41	*c*.6067G > A	*p*.G2023R	35,83	Mutation uS
	7	*c*.800C > T	*p*.T267I	32,99	Bisher unbekannt
	7	*c*.828A > C	*p*.K276N	15,49	Bisher unbekannt
BRCA 1	11	*c*.3700_3704	*p*.V1234Qfs8	45,75	**Pathogen, Kat 5**
	16	*c*.4676-1G > A	*p*.4676-1G > A	11,94	**Pathogen, Kat 5**
	2	*c*.65_69delAG	*p*.E23Vfs17	85,13	**Pathogen, Kat 5**
BRCA 2	10	*c*.1241dup	*p*.L414FFs7	34,35	Unbekannt, aber trunkiert ≥ *pathogen*
	10	*c*.1273G > T	*p*.E425	22,31	Unbekannt, aber trunkiert ≥ *pathogen*
	11	*c*.2100delA	*p*.700FFs30	76	**Pathogen, Kat 4**
	11	*c*.2957_2958insA	*p*.N986Kfs2	28,89	**Pathogen, Kat 5**
	11	*c*.3847_3848delGT	*p*.V1283Kfs2	79,39	**Pathogen, Kat 5**
	11	*c*.3680delA	*p*.N128lfs6	38,43	**Pathogen, Kat 5**
	11	*c*.3962_4066delinsTAAA	*p*.D1321Vfs6	47,47	Unbekannt, aber trunkiert ≥ *pathogen*
	11	*c*.6486_6489delACAA	*p*.K2162Nfs5	18,61	**Pathogen, Kat 5**
	13	*c*.7007 + 2T > A	*c*.7007 + 2T > A	58,21	**Pathogen, Kat 5**
	17	*c*.7883T > C	*p*.I2628T	69,28	Kategorie 3
	25	*c*.9284A > G	*p*.D3095G	77,89	Kategorie 3
	3	*c*.241T > A	*p*.F81I	53,74	Kategorie 3
	4	*c*.347_349delinsT	*p*.S116lgs7	15,01	Unbekannt, aber *trunkiert* ≥ *pathogen*
CDK12	1	*c*.469G > T	*p*. (Glu175TER)	15,54	*Trunkiert* ≥ *pathogen*
	14	*c*.4267G > A	*p*.A1423T	46,4	Bisher unbekannt
CHEK1	7	*c*.767del	*p*.T226Hfs14	10,7	*Trunkiert* ≥ *pathogen*
CHEK2	–	–	–	–	–
FANCA	22	*c*.1916T > C	*p*.Val639Ala	44,2	Bisher unbekannt
RAD51C	2	*c*.262C > A	*p*.L88M	11,24	Bisher unbekannt
	5	*c*.730A > G	*p*.I244V	50,3	MuS
	5	*c*.748C > T	*p*.H250Y	43,26	Bisher unbekannt
	5	*c*.790G > A	*p*.G264S	33,63	MuS
HRD-Score	95,1–175,2 (positiv > 50)				Pathogen

*fett* therapeutischer Effekt von PARPi gesichert, *kursiv* Therapieeffekt von PARPi wahrscheinlich, *MuS* Mutation unbekannter Signifikanz

Mit einem PARP-Inhibitor wurden 12/17 Patienten mit einer BRCA 1/2 Mutation therapiert. Es wurde ein PSA-Ansprechen in 67 % (8/12) der Patienten erzielt; das mediane progressionsfreie Intervall bis zum erneuten PSA-Anstieg betrug 9,8 (2,3–27,4) Monate.

Mit Olaparib wurden 5/8 Patienten mit einem positiven HRD-Score behandelt und zeigten ein PSA-Ansprechen ≥ 50 % in 80 % sowie ein biochemisches rezidivfreies Überleben von 10,4 (5,2–28,1) Monaten.

### Androgenrezeptormutationen

Mutationen des AR fanden sich bei 42 (14,1 %) Patienten. Alle Mutationen waren dabei in der ligandenbindenden Domäne (LBD) lokalisiert und hatten unterschiedliche therapeutische Implikationen zur Folge (Tab. 6). 5 Patienten wiesen multiple Mutationen in der LBD des AR auf und weitere 3 Patienten zeigten neben der AR-Mutationen begleitende Alterationen zusätzlicher Gene (Tab. 7). Die überwiegende Mehrzahl der Mutationen waren solche, die eine Resistenz gegen die klassischen nicht steroidalen Antiandrogene wie Bicalutamid oder Flutamid bzw. eine Resistenz gegen Abirateron induzierten. Nur eine Mutation (V716M) bedingt eine agonistische Wirkung von Darolutamid, während die anderen Mutationen meist zu einer agonistischen bzw. partiell agonistischen Wirkung von Apalutamid bzw. Enzalutamid führten. Die Mutation T878A ist von daher interessant, da diese zu einer agonistischen Wirkung von Prednison führt, aber eine antagonistische Wirkung von Dexamethason induziert.

**Tab. 6:** AR-Mutationen und deren therapeutische Implikation

Gen	Mutation	NG Change-Protein	NG Allelfrequenz	Funktion
AR	*c*.1382_1417del	*p*.G461_G473	94,07	Unbekannt
	*c*.2105T > A	*p*.L702H	14,7	Resistenz nach Bic, Abi; Apa, Enza, Daro als Antagonist
	*c*.2623C > T	*p*.875Y	14,12	Apa, Enza als Agonist, Östradiol aktiviert, Darolutamid als Antagonist
	*c*.2632A > G	*p*.T878A	69,34	Apa, Bic, Enza, Flu als partieller Agonist, Abi + Pred als Agonist, Darolutamid als Antagonist, Abi + Dexa als Antagonist
	*c*.2671G > C	*p*.D891H	90,97	Unbekannt
	*c*.2395C > G	*p*.Q799E	99,93	Unbekannt
	*c*.2146G > A	*p*.V716M	51,69	Darolutamid als Agonist; Resistenz Abi und Bica; Enza als Antagonist
	*c*.1937C > A	*p*.A646D	100	Funktionsverlust
	–	W742W/L	–	Resistenz nach Bic, Flu; Apa, Enza, Daro als Antagonist
	–	F877L	–	Apa, Enza part. Agonist, Bic, Daro als Antagonist
	–	T878S	–	Apa, Bic, Enza, Flu als part. Agonisten
AR-Fusion	AR:NLGN1	chrX:66931466:chr3:17320306	–	Unbekannt, in der Literatur zeigen Fusionen Resistenzen zu allen ARTA
AR, multipel	*c*.2105T > A	*p*.L702H	24,04	Resistenz Bic, ABi
	c.2623C > T	p.875Y	23,4	Apa, Enza als Agonist, Daro als Antagonist
	*c*.2105T > A	*p*.L702H	3,16	Resistenz Bic, Abi
	c.2623C > T	p.874Y	4,49	Apa, Enza Agonist, Daro Antagonist
	–	–	–	Apa, Bic, Enza, Flu als partieller Agonist
	c.2632A > G	p.T878A	49,79	Abi + Pred als Agonist Darolutamid als Antagonist Abi + Dexa als Antagonist
	*c*.2623C > T	*p*.875Y	88,27	Apa, Enza als Agonist, Daro als Antagonist
	AR-V7	–	–	Resistenz gegen Abi, Enza
	*c*.2105T > A	*p*.L702H	48,15	Resistenz Bic, Abi, Steroide
	c.2632A > G	p.T878A	47,51	Apa, Enza als Agonist, Daro als Antagonist

**Tab. 7:** Mutationen des p53-Gens

Gen	Exon	Mutation	NG Change-Protein	NG Allelfrequenz	Funktion
P53	4	*c*.2480A > G	*p*.K827R	90,97	Bisher unbekannt
	5	*c*.3153 + 3G > T	*c.*3153 + 3G > T	8,23	Mutation in Splice site, Funktion unbekannt
	–	*c*.396G > C	*p*.K132N	5,09	Nicht funktionelles Protein
	6	*c*.584T > C	*p*.I195T	18,61	Nicht funktionelles Protein
	7^*^	*c*.742C > T	*p*.R248W	85,07	„Gain of function“, Resistenz gegenüber Chemotherapie
	–	*c*.763A > T	*p*.I255F	5,2	Funktionsverlust
	–	*c*.743G > A	*p*.R248Q	63,2	Nicht funktionelles Protein
	8	*c*.916C > T	*p*.R306^a^	50,49	Trunkiertes Protein, wahrscheinlich Funktionsverlust
	–	*c*.817C > T	*p*.R273H	86,79	„Gain of function“, dadurch Inaktivierung von ATM
	10	*c*.1009C > T	*p*.R337C	51,7	Nicht funktionelles Protein
	17	*c*.488A > G	*p*.Tyr163Cys	45,6	Nicht funktionelles Protein

^a^Diese Mutation wurde bei 2 Patienten identifiziert

Es erfolgte bei 12 Patienten mit einer AR-Mutation L702H und W742L eine Umstellung auf Apalutamid oder Enzalutamid. Es wurde ein PSA-Ansprechen bei 9 Patienten für im Mittel 6,5 (3–15) Monate erreicht. Eine Umstellung auf Darolutamid ist aufgrund der Zulassungssituation bei keinem Patienten vorgenommen worden. Bei 2 Patienten mit einer T878A-Mutation wurde eine Umstellung auf Dexamethason vorgenommen. Beide Patienten erreichten eine PSA-Reduktion > 50 % für 6 und 8 Monate.

### P53-Mutationen

Mutationen des p53-Tumorsuppressorgens wiesen 66 (22,1 %) Patienten auf, von denen 90 % in der DNA-bindenden Domäne gelegen waren (Tab. 7). Nahezu alle diese Mutationen resultieren in einem Funktionsverlust des p53, während 3 Mutationen in den Exons 5–8 mit einem sog. „gain of function“ assoziiert waren. Eine Mutation fand sich in der Oligomerisationsdomäne in Exon 10 und führt zu einem Funktionsverlust. 2 weitere Mutationen waren in den für die Funktion des Tumorsuppressorgens bedeutungslosen Exons 4 bzw. 11 lokalisiert.

Für die Mutationen in der DNA-bindenden Domäne stehen aktuell keine spezifischen therapeutischen Ansätze zur Verfügung. Für die Patienten mit einer Gain-of-function-Mutation könnten theoretisch PARP-Inhibitoren eingesetzt werden. Aufgrund der Ablehnung eines Kostenübernahmeantrages haben wir diesen Ansatz bei unseren Patienten jedoch nicht umsetzen können.

### PIK3CA/PTEN

Mutationen im PIK3CA-Pathway bzw. ein Verlust von PTEN wurde bei 19 Patienten identifiziert. Hier handelte es sich in der überwiegenden Mehrzahl um aktivierende Mutationen des PIK3Ca bzw. um einen Funktionsverlust von PTEN (Tab. 8).

Es erfolgte keine gezielte Therapie mit dem AKT-Inhibitor Ipatasertib.

**Tab. 8:** Mutationen des PIK3CA und des Wnt-Pathways

Gen	Exon	Mutation	NG Change-Protein	NG Allelfrequenz	Funktion
PIK3CA	2	*c*.263G > A	*p*.R88Q	11,88	Wahrscheinlich aktivierend
	9	*c*.1571G > A	*p*.R524K	4,95	Funktion unbekannt
	10	*c*.1633G > A	*p*.E545K	23,83	Aktivierend
PTEN	5	*c*.405DUP	*p*.C136Mfs × 44	60,77	Trunkiertes Protein
	7	*c*.752G > C	*p*.G251A	14,03	Funktion unbekannt
	8	*c*.923G > A	*p*.R308H	14,79	Funktion unbekannt
*Wnt-Pathway*					
CTNNB1	3	*c*.100G > A	*p*.G34R	18,41	Andere Mutationen in dem Codon sind aktivierend
	3	*c*.101G > A	*p*.34E	23,28	Aktivierend
	3	*c*.110C > G	*p*.537C	25,57	Aktivierend
	3	*c*.133T > C	*p*.545P	31,84	Aktivierend
	3	*c*.94G > A	*p*.D32N	5,5	Aktivierend
	3	*c*.94G > T	*p*.D32Y	8,77	Wahrscheinlich aktivierend

### Wnt-Pathway

Aktivierende Mutationen im Wnt-Pathway fanden sich bei 12 Patienten, wobei aktivierende Mutationen für CTNNB1 im Vordergrund stehen (Tab. 8).

Bei fehlenden Therapieoptionen konnte keine zielgerichtete Therapie eingeleitet worden.

### Seltene Mutationen

Insgesamt 27 Patienten zeigten seltene isolierte Mutationen einzelner Gene oder eine Kombination von seltenen Mutationen (Tab. 9). Zielführend wurde bei 3 Patienten eine Therapie mit Pembrolizumab bei MSI-high bzw. MSH6-Verlust eingeleitet, die ein PSA-Ansprechen ≥ 50 % induzierte, das für 4, 6 und 8 Monate anhielt.

**Tab. 9:** Mehrfachmutationen und potenzielle therapeutische Implikationen

Mutation	Wirkung	Therap. Implikation
AR_L702H_ + CDK12 + PTEN + p53	Resistenz Bicalutamid	*ARTA* *+* *Ipatasertib*
	Zellzyklusproliferation↑	**SR-4835 (DDR-Proteine↓)** + *PARPi*
TRPMS/ERG + CHEK2	CHEK2 ≥ Antigenpräsentation↑, STING pathway Aktivierung und PD-L1 Expression↑	*PD-L1 Inhibitor* (Glioblastom)
APC +	Kolorektale Karzinome	*Statine*
p53 +	Zellzyklus↑	**Undruggable**
DNMT3A +	Prognostisch bei AML ≥ Chemoresistenz	*5‑Azacytidin*
AR:NLGN1	NLGN1 ≥ Schizophrenie, Autismus	**Undruggable**
NKX3-1 +	TSG ≥ PCA Initiierung, CRPC	**Undruggable**
PIK3CA +	Aktivierung AKT Pathway	–
–	Zellzyklus↑	*Ipatasertib*
HDAC2 +	Häufig in MSI-high CRC	*evtl. Pembrolizumab*
CHEK 1	Zellzyklusprogression	**Undruggable**
BMP1 +	Mesenchymzelle ≥ Osteoblast	**Undruggable**
CHEK1 + 2 +	Zellzyklusprogression	*evtl. PD-L1 Inhibitor*
PTEN +	Zellteilung, -wachstum, -differenzierung	**Undruggable**
p53	Zellzyklusprogression	**Undruggable**
HNF1a +	Transkriptionsfaktor	*Gemcitabin*
RNF43 +	Regulation Wnt-Pathway, CRC, Endometriumkarzinom	**Assoziation mit BRAFV600**^**mut**^ ≥ **z.** **B. Encorafenib-Cetuximab**
SF3B1	Splicing-Faktor RNA-Synthese, DNA-Reparatur	*PARPi*
BRCA 2 +	DNA-Reparatur	*PARPi*
MSH6 +	DNA-Stabilität	*Pembrolizumab*
PMS2	DNA-Stabilität	*Pembrolizumab*
PIK3CA +	Regulation Wachstum	*Ipatasertib*
PTEN +	Proliferation und Stoffwechsel	–
–	Regulation Zellteilung, -wachstum, -differenzierung	–
AR_L702H_	Zelluläre Kontrolle Prostata	*Apa, Daro, Enza*
*β*-Catenin +	Transkriptionsfaktor	**Undruggable**
PIK3CA +	Regulation Wachstum	*Ipatasertib*
p53 +	Proliferation, -stoffwechsel Zellzyklusprogression	–
MSH6	DNA-Stabilität	*Pembrolizumab*

*kursiv* in der Literatur beschriebenes Medikament zur zielgerichteten Therapie, *fett* keine Therapie verfügbar

Mehrfachmutationen wiesen 9 (3,1 %) Patienten in 3 (*n* = 4) bzw. 4 (*n* = 5) verschiedenen Genen auf (Tab. 7). Von den 31 nachgewiesenen Mutationen wäre für 17 (54,8 %) potenziell eine zielgerichtete Therapie denkbar, die wir jedoch nur bei Patient 7 umgesetzt haben. Die Kombination von Pembrolizumab und Olaparib resultierte in einem medianen progressionsfreien Überleben von 17,5 Monaten.

## Diskussion

Die Kombination einer Androgendeprivation mit einem ARI stellt die Therapie der Wahl des mHSPC dar [3, 4]. Bei Eignung der Patienten für eine Docetaxel-Therapie sollte die Triple-Therapie durch die Addition von Abirateron/Prednison oder Darolutamid insbesondere bei hoher Metastasenlast angewendet werden. [12, 13]. Trotz des erheblichen Benefits in Bezug auf das radiologische progressionsfreie Überleben (rPFS) und das Gesamtüberleben werden auch in Deutschland noch immer nur ca. 40 % der mHSPC-Patienten leitliniengerecht behandelt [14]. Mehr als der Hälfte der Patienten wird eine der lebensverlängernden Therapien aus nicht nachvollziehbaren Gründen vorenthalten.

Trotz eines initial guten therapeutischen Ansprechens entwickeln nahezu alle mHSPC-Patienten eine Resistenz auf die alleinige oder kombinierte Hormontherapie, so dass nachfolgende Zweit- und Drittlinientherapien mit alternativem Wirkmechanismus (zytotoxische oder Radioligandentherapie, PARP-Inhibitoren) eingesetzt werden, um ein erneutes Ansprechen zu induzieren [2]. Letztendlich ist keine der innovativen Therapien kurativ oder von langer Wirkdauer, so dass es sinnvoll erscheint, mit den modernen Methoden der molekularpathologischen Diagnostik frühzeitig Treibermutationen zu identifizieren, die in den Prozess der Resistenzentwicklung und Tumorprogression involviert sind und die eventuell durch eine zielgerichtete medikamentöse Therapie inhibiert werden können [10].

Die überwiegende Mehrzahl der Patienten entwickelt die Androgenresistenz bereits im Progress nach der ersten kombinierten Hormontherapie [15]. In dieser Situation besteht eine Zulassung für die Anwendung von PARP-Inhibitoren bei Nachweis einer BRCA 1/2-Mutation [16]. Auch wenn die PARP-Inhibitoren auf dem Boden der aktuellen Zulassungssituation auch bei fehlendem Nachweis einer BRCA-Mutation angewendet werden können, zeigen die Subgruppenanalysen der Studien einen klinisch relevanten Benefit letztendlich nur für die Patienten mit Mutationen der BRCA-Gene [18, 19]. Wir favorisieren deshalb, die molekulare Testung bereits beim ersten Übergang des mHSPC in das mCRPC, spätestens jedoch nach Versagen der ersten Zweitlinientherapie.

Die Ergebnisse unserer retrospektiven Analyse zeigen, dass eine molekulare Testung von Gewebeproben aus archiviertem Material oder frisch gewonnenen Biopsien progredienter Metastasen unproblematisch nach interdisziplinärer Diskussion in den klinischen Alltag integriert werden kann. Wir können zeigen, dass bei zwei Dritteln der Patienten Metastasen vorliegen, die meist CT-gestützt, seltener sonographisch gestützt biopsiert werden können und bei denen unabhängig von der Lokalisation der Metastasen in 85 % (Knochen) bis 95 % (Weichteilmetastasen) ausreichende DNA-Mengen zur molekularen Analyse extrahieren werden können. Gerade bezüglich der hohen Verwertbarkeit auch der Biopsien aus ossären Metastasen spielen die modernen Methoden des „next generation sequencing“ (NGS) aufgrund der gegenüber früheren Techniken zur validen Auswertung deutlich geringeren Menge an DNA eine entscheidende Rolle [10]. Wir favorisieren bezüglich der Mutationsanalyse eine Biopsie aus den in der bildgebenden Diagnostik nachweisbaren progredienten Metastasen, da sich hier am ehesten die für die Progression verantwortlichen somatischen Mutationen detektieren lassen. Auf das archivierte Gewebe greifen wir nur dann zurück, wenn die Metastasen nicht biopsierbar sind bzw. keine ausreichende DNA-Menge aus der Biopsie extrahiert werden kann. In diesem Zusammenhang konnten Hussain et al. [11] zeigen, dass die Ausbeute der NGS umso höher ist, je besser das zur Untersuchung zur Verfügung stehende Biomaterial ist und stützen damit unsere Strategie der Biopsie progredienter Metastasen. Als Alternative zur molekularen Analyse von Gewebeproben steht prinzipiell auch zirkulierende Tumor-DNA (ctDNA) zur Verfügung. Diesbezüglich konnte in einer Auswertung der PROfound-Studie gezeigt werden, dass nur eine geringe Konkordanz von 27 % bezüglich des Vorliegens von homozygoten Deletionen für BRCA und ATM zwischen ctDNA und Gewebe vorhanden ist, während sich eine hohe Konkordanz für Nonsense‑, Splice- und Frameshift-Mutationen zeigte [21]. Wir favorisieren aus diesem Grund derzeit weiterhin die NGS-Analyse aus Gewebeproben biopsierter Metastasen.

Wir haben mit unserer retrospektiven Analyse auch zeigen können, dass ein bezüglich der häufigsten beim mCRPC vorkommenden Mutationen zusammengestelltes Panel in nahezu 50 % der Patienten eine therapierbare Mutation erkennt und dass die generelle Anwendung eines TSO500-Panels nicht zielführend und kostenorientiert ist. Nur bei einer minimalen Anzahl von Patienten können durch das ausgedehnte TSO500-Panel zusätzliche potenziell therapierbare Mutationen identifiziert werden.

Bezüglich der identifizierten Mutationen waren Alterationen der BRCAness-Gene, des p53 Tumorsupressorgens und des Androgenrezeptors mit 22 % bzw. 14 % am häufigsten nachweisbar und decken sich mit den Daten anderer klinischer Studien [10, 11, 15, 21, 30]. Die überwiegende Mehrzahl der Mutationen des p53 kann aktuell noch nicht therapiert werden bzw. die theoretisch zur Verfügung stehende Therapieoption mit wee-1 Kinase-Inhibitoren ist aufgrund der hohen therapieassoziierten Toxizitäten noch nicht in den klinischen Alltag integriert. [23]. Nur die 3 von uns beschriebenen Mutationen mit dem „gain of function“ könnten prinzipiell therapeutisch genutzt werden. Die Mutationen p53^R248W^ sowie p53^R273H^ führen zu Konformationsveränderungen des p53 mit einem daraus resultierenden Verlust der Tumorsuppressoraktivität [24]. In präklinischen Untersuchungen konnte gezeigt werden, dass diese beiden Mutationen nach Schädigung der DNA durch γ‑Strahlung zu einer Verlagerung des MRN-Komplex an die Position der geschädigten DNA führt und dadurch eine Inaktivierung des ATM mit konsekutiver genetischer Instabilität bedingt. In dieser klinischen seltenen Situation könnte ein PARP-Inhibitor ein therapeutisches Ansprechen erzielen. Des Weiteren sind die beschriebenen GOF-Mutationen mit einem verkürzten Überleben bei verschiedenen Tumorentitäten sowie mit einer Chemoresistenz gegenüber Zystostatika durch eine Induktion von CYP3A4 assoziiert [25].

Für die Mutationen der BRCAness-Gene und der AR-Mutationen stehen klinisch anwendbare Therapieoptionen zur Verfügung [16–20, 26]. Niraparib, Olaparib, Rucabarib und Talazoparib haben in prospektiv randomisierten Studien eine klinisch bedeutsame therapeutische Wirksamkeit entfaltet, wenngleich besondere Aspekte der Wirksamkeit berücksichtigt werden müssen [16–20, 26]. In der PROfound-Studie war der signifikante Benefit von Olaparib nur für die Patienten mit einer BRCA 1/2- oder ATM-Mutation nachweisbar, jedoch nicht für die Patienten mit anderen Mutationen [16]. In der TRITON-Studie wurde gezeigt, dass Rucaparib nur bei Patienten mit einer BRCA 1/2-Mutation einen Benefit erzielt, nicht jedoch in der ATM-Subgruppe [26]. Die TALAPRO-Studie zeigte eine hohe objektive Ansprechrate von 81,5, 81,8 und 70 % für Mutationen von BRCA 1/2, ATM bzw. CDK12, während Mutationen von PALB‑2 und CHECK2 nur mit einer Ansprechrate von 33 % bzw. 50 % assoziiert waren [20]. In der Magnitude-Studie hingegen wurde ein Benefit in Bezug auf das rPFS sowohl für die BRCA-Gruppe als auch für die Gruppe mit anderen Mutationen der HRR-Gene dokumentiert [17]. Diese Daten der verschiedenen Studien verdeutlichen, dass im klinischen Alltag eine fundierte molekulare Analyse der gesamten Bandbreite der BRCA-Gene erforderlich ist, um eine individuelle Entscheidung für den einen oder anderen PARP-Inhibitor zu treffen.

Beim Ovarialkarzinom und auch beim Mammakarzinom hat sich neben der Testung auf eine Mutation von BRCA 1/2 der HRD-Score als allgemeiner Parameter der Defizienz einer homologen Rekombination durchgesetzt. Der HRD-Score („homologous recombination deficiency“) setzt sich aus drei unabhängigen Markern („loss of heterozygosity“ [LOH], „large scale state transitions“ [LST] und „telomeric allelic imblance“ [TAI]) zusammen und kennzeichnet eine gestörte homologe Rekombination, die ein positives Ansprechen auf PARP-Inhibitoren signalisiert [27]. Um zwischen HRD-Positivität bzw. HRD-Negativität zu unterscheiden, werden in der Labordiagnostik klinisch validierte Schwellenwerte für die jeweiligen HRD-Scores definiert. Wie in unserer Studie gezeigt, können eine Vielzahl von inaktivierenden Mutationen der HRD-Gene beim mCRPC durch NGS-Verfahren identifiziert werden. Beim Ovarialkarzinom wurde für Olaparib die Zulassung bei positivem HRD-Score ausgesprochen, nachdem sich ähnlich für BRCA 1/2 Mutationen ein erheblicher Überlebensbenefit darstellen ließ [28]. Auch wenn es beim Prostatakarzinom noch keine entsprechenden klinischen Studien zum HRD-Score gibt, haben wir diesen Marker in die routinemäßige molekulare Analyse integriert. Patienten mit positivem HRD-Score, aber fehlender BRCA 1/2-Mutation werden mit einem PARP-Inhibitor behandelt.

Die molekulare Analyse des Androgenrezeptors (AR) erbrachte bei 14 % der Patienten Mutationen mit therapeutischer Implikation. Die Mutationen des AR sind als ein durch die vorhergehende ADT in Kombination mit ARIs induzierter Resistenzmechanismus anzusehen, der sich bei der Mehrzahl der von uns untersuchten Patienten schon nach der antihormonellen Erstlinientherapie entwickelt [15, 30–32]. So haben wir keine signifikanten Differenzen in der Häufigkeit und der Art der AR-Mutationen bei Patienten identifiziert, die unmittelbar nach Versagen der Erstlinientherapie oder erst nach Versagen auch einer Drittlinientherapie molekular getestet worden sind. Alle Mutation wurden in der LBD des AR identifiziert und wirken sich dahingehend aus, dass die eingesetzten AR-blockierenden Medikamente teils als totale oder als partielle Agonisten wirken und somit den Progress der Erkrankung beschleunigen (Tab. 6). Bei den meisten AR-Mutationen wirken die klassischen ARIs Abirateron, Apalutamid bzw. Enzalutamid agonistisch, während Darolutamid als Antagonist in diesen Situationen eingesetzt werden kann [30, 32]. Nur bei der AR-Mutation V716M wirkt Darolumatid agonistisch und die anderen ARI sind antagonistisch wirksam [29]. Andere Mutationen (L702H, W742L, T878A) induzieren einen promiskuitiven AR, der unter Prednison/Prednisolon eine Proliferationsstimulation bewirkt, während die Applikation von Dexamethason eine Proliferationsinhibierung induziert. Diese Daten verdeutlichen aus unserer Sicht, dass eine fundierte molekulare Analyse des AR nach Versagen der initialen antihormonellen Kombinationstherapie erworbene Resistenzmechanismen aufzeigen kann, die durch eine einfache Umstellung der ARI-Komponente oder im Falle von Abirateron/Prednison mit dem Ersatz des Prednison durch Dexamethason ein weitergehendes therapeutisches Ansprechen induzieren kann. Aus den Daten der hier vorliegenden Arbeit sowie aus den bereits publizierten Daten möchten wir deshalb empfehlen, eine molekulare Analyse auf das Vorliegen von AR-Mutationen sowie Mutationen der HRD-Gene nach Versagen der Erstlinientherapie in den klinischen Alltag zu integrieren. Wir gehen aktuell so vor, dass wir bei entsprechender AR-Mutation eine Umstellung der Therapie vornehmen und dadurch noch ein mittleres therapeutisches progressionsfreies Intervall von 6–8 Monaten induzieren können.

In unserem molekularen PCA-Panel haben wir die Untersuchung auf eine Mikrosatelliteninstabilität (MSI) sowie den Verlust der Mismatch-repair-Gene (MMR) MLH1, MSH6, MSH, MSH2, PMS2 integriert, da sich aus dieser einfachen immunhistochemischen Analyse wichtige therapeutische Implikationen für eine geringe Anzahl von Patienten ergeben könnten. Zirka 2 % der von uns getesteten Patienten zeigten eine entsprechende Mutation, was sich mit den Daten der Literatur deckt, in der diese Alterationen in 3–5 % der mCRPC-Patienten beschrieben werden [33]. Lediglich beim duktalen PCA wird eine Mutationsfrequenz von bis zu 40 % angegeben, so dass bei diesen Patienten ein generelles Screening bereits bei Erstdiagnose überlegt werden kann [34]. Sowohl für die Situation der MSI-high als auch für den Verlust der MMR-Gene kann Pembrolizumab als therapeutische Option appliziert werden, wenn keine anderen vertretbaren Möglichkeiten bestehen. Ein therapeutisches Ansprechen ist bei > 50 % der Patienten zu erwarten [35]. Auch in unserem Patientenkollektiv zeigte sich ein PSA-Ansprechen bei 2 von 3 Patienten für ein mittleres Zeitintervall von 6 Monaten. Auch wenn der Verlust der MMR bzw. eine MSI-high nur bei knapp 2 % der Patienten nachweisbar war, sollte die einfache Untersuchung in das diagnostische Armentarium zumindest nach Versagen der Zweit- oder Drittlinientherapie integriert werden.

Eine Mutation für PIK3CA bzw. einen Verlust von PTEN wiesen 8 % der Patienten auf, die uns weiterhin vor eine therapeutische Herausforderung stellt. Prinzipiell könnte hier die Therapie mit dem AKT-Inhibitor Ipatasertib im Sinne einer Off-label-Anwendung erwogen werden. Jedoch zeigen die Daten der IPATential150-Studie ein marginales Ansprechen bei hoher therapieassoziierter Nebenwirkungsrate [36]. In die prospektiv randomisierte klinische Phase-III-Studie wurden 1101 Patienten mit einem mCRPC in die beiden Therapiearme Abirateron/Prednison plus Ipatasertib vs. Abirateron/Prednison mit dem Ziel der Verbesserung des rPFS randomisiert. Nach einem medianen Follow-up von 19 Monaten (0–33) war ein statistisch signifikanter, klinisch jedoch moderater Benefit im rPFS von 16,5 vs. 18,5 Monaten (HR 0,77, 95 %-CI 0,61–0,98, *p* = 0,034) zu verzeichnen. Jedoch zeigten sich in der Gruppe der Kombinationstherapie ≥ Grad-3-Nebenwirkungen in 70 % vs. 39 % sowie eine deutlich höhere Rate an frühzeitigem Therapieabbruch von 21 % gegenüber 5 %. Auch zeigen die aktualisierten Daten bei längerem Follow-up keinen Benefit des Gesamtüberlebens, so dass sich derzeit für Patienten mit einer Aktivierung des PIK3CA/AKT-Pathways bzw. einem PTEN-Verlust keine wirklich guten Therapiemöglichkeiten ergeben. Es könnte lediglich im Sinne einer Off-label-Anwendung die Kombination von Ipatasertib mit einem anderen ARTA in Abhängigkeit des AR-Mutationsstatus individuell angewendet werden. Zu diesem Ansatz zeigt eine aktuelle Phase-Ib-Studie der Kombination Ipatasertib (4 × 400 mg/Tag) plus Darolutamid (2 × 600 mg/Tag) eine minimale Interaktion der beiden Medikamente, eine Rate an ≥ Grad-3-Nebenwirkungen und frühzeitigen Therapieabbrüchen bei nur 14 % bzw. 7 % der Patienten [37]. Wir haben keine Erfahrungen in der Anwendung von Ipatasertib und wären aufgrund der beschriebenen Datenlage eher zurückhaltend in der Anwendung dieser Therapieoption.

Mutationen im Wnt-Pathway zeigten 5 % unserer Patienten, die aktuell aufgrund fehlender medikamentöser Ansatzpunkte zwar noch nicht therapeutisch genutzt werden können, die aber Hinweise auf mögliche Resistenzmechanismen gegenüber ARI geben. So konnte in verschiedenen Studien gezeigt werden, dass die Aktivierung des Wnt-Pathways zu einer Resistenz gegenüber Abirateron führt und somit zur individuellen Therapieplanung genutzt werden könnte [38].

## Fazit für die Praxis

- Die molekulare Analyse mittels NGS-Technik bei 311 mCRPC-Patienten zeigt, dass unabhängig von der Lokalisation der Gewebeproben eine DNA-Ausbeute von 85–95 % erreicht werden kann.
- In dem gesamten Kollektiv wiesen nur 25 % der Patienten einen Wildtyp auf, während sich bei 75 % Mutationen unterschiedlicher klinischer Relevanz zeigten.
- Knapp 50 % der Patienten wiesen Mutationen auf, die sich therapeutisch durch eine Umstellung der ADT, durch die Addition eines PARP-Inhibitors oder durch die zusätzliche Anwendung eines AKT-Inhibitors bzw. eines PD-L1-Inhibitors nutzen ließen.
- Eine erste Analyse der AR- und der BRCA-Mutationen sollte nach Versagen der Erstlinientherapie erfolgen, da sich unmittelbare Therapieoptionen mit bereits zugelassenen Medikamenten ergeben.
- Eine ausgedehnte Panelanalyse empfiehlt sich nach Versagen der Zweit- oder Drittlinientherapie.
- Seltene Mutationen oder Kombinationen von Mutationen sollten zur weiteren Therapieplanung immer in einem molekularen Tumorboard diskutiert werden.

## Data Availability

Die erhobenen Datensätze können auf begründete Anfrage in anonymisierter Form beim korrespondierenden Autor angefordert werden. Die Daten befinden sich auf einem Datenspeicher an der Klinik für Urologie der Uniklinik Köln.
